# Notes and News

**Published:** 1888-02-04

**Authors:** 


### Notes and News.

Collected and Communicated.

The next evening meeting of The Hospitals Association will be held in the Board-room of St. George's Hospital, Hyde Park Corner, S.W., on Wednesday, February 8th, at eight p.m., when Mr. Timothy Holmes, F.R.C.S., will read a paper on "The Relation of the Medical School to the Hospital." Dr. Bristowe, F.R.S. (President) will preside. All members of the medical profession and others interested in this question are cordially invited. Cards can be obtained on application from Mr. Howard J. Collins, Secretary, The Hospitals Association, Norfolk House, Norfolk Street, W.C.

Mr. G. Lund and Mr. Christopher Swift have written to the local newspapers, to express their thanks to the officials of the Mexboro' Fever Hospital and the Swinton Small-pox Hospital respectively for the care and kindness they have received in these institutions. This is a graceful and courteous thought which might well be imitated by other patients.

At the annual meeting of the Jenny Lind Hospital the Mayor appealed to the public for means to extend the means and usefulness of this beneficent institution, and the Dean expressed his conviction that the spirit of the noble and talented woman who founded the institution was still, though she was removed from earthly sight, deeply interested in its work.

Let the oft-maligned medical student, the object of so much cheap and unmerited satire, have his due. The entire cost of the Christmas entertainment at King's College Hospital was borne by the students attending it. The only thing to be regretted in this matter is that the students stepped into the breach because the funds of the hospital are so low that the authorities did not feel justified in spending any on an entertainment for the patients.

Dr. H. Burnes, of Upper Holloway, one of the governors of the Great Northern Central Hospital, has issued a circular to the authorities of the various hospitals suggesting a central committee, who should appoint a superintendent almoner and assistants to inquire into the pecuniary circumstances of all applicants for relief as out-patients. Dr. Burnes states that he personally knows of well-to-do tradesmen getting costly surgical appliances at reduced cost, of patients getting drugs and selling them to chemists, and of other abuses which demand investigation and suppression.

The doctors of the Charité Hospital, Paris, have presented an address to the Augustinian nuns who are now being dismissed from that hospital, to be replaced by lay nurses, in accordance with a municipal decree. In it they express the most flattering opinion of the "Sœurs Augustines" as nurses, and they are said to regret deeply that the money now to be expended in salaries to the new nurses, who cannot, like the Sisters, give their services gratis, has not been used to provide more beds in the hospital wards. Each nun was presented with a bouquet by the patients as she left the hospital. The nuns now at the Hotel Dieu and the St. Louis Hospital are soon to be dismissed also.

The medical men of Lincoln who are unconnected with the hospital have recently passed a resolution recommending an increase of the medical staff of the hospital. The repairs and building works which have taken place during the year in connexion with the institution have cost £420.

A more excellent memorial of the Queen's Jubilee having been commemorated within the Abbey precincts of Westminster, it would be difficult to find than the endowment of three cots in the Westminster Hospital. The cost of endowment was £1,800, of which Mr. James Hora, of Victoria Street, gave £600, the Baroness Burdett-Coutts being among the other contributors. The children of poor persons residing in the parishes of St. Margaret and St. John the Evangelist are to have a preference of admission to these cots.

A new institution has been added to the charities of Hull. This is the "Hull and East Riding and North Lincolnshire Orthopædic Hospital." The institution has been established on a very modest basis, but from the good work it is doing in curing those who might, without timely attention, become cripples for life, it deserves the support of the charitable public. The surgeon to the hospital is Dr. Hagyard, who up to the present time has borne nearly all the cost of maintaining it.

At the annual meeting of contributors to the Edinburgh Sick Children's Hospital it was stated that the subscriptions for the year amounted to £2,731 6*s*. 10*d*., the donations to £947 3*s*. 9*d*., and the interest on investments to £189 14*s*. 10*d*., making, with £57 10*s*. 3*d*. of incidental receipts, the total ordinary income £3,925 15*s*. 9*d*. The expenditure for the year amounted to £2,875 14*s*. 6½*d*. This is the first time the income has met the expenditure, and it is hoped that this satisfactory condition of affairs will go on to even better things. A ward to be exclusively devoted to surgical cases was opened on November 1st, under the superintendence of Mr. Joseph Bell, President of the Royal College of Surgeons, who has kindly promised to give his services as surgeon.

The National Hospital for the Paralysed and Epileptic, Queen Square, Bloomsbury, occupies an altogether exceptional position. Those who are received within its walls are, as a rule, ineligible for other hospitals, require long and careful treatment, and may in some cases be regarded as all but incurable. If they cannot be cured, or at least greatly relieved, they are doomed to perpetual helplessness and dependance on others, even when they are so fortunate as to escape terrible pain, which often results in loss of intellect. It is one of the misfortunes attendant on such a civilisation as ours that its haste and intellectual activity are so apt to induce nervous diseases of every sort. Sometimes these are congenital, but in many cases they are the direct result of that mental overstrain from which our best and most thoughtful often suffer. This being so, that hospital has a strong claim on our consideration which at once gives shelter and relief to those already afflicted, and affords our students and doctors the opportunity for studying a class of diseases of the most important and mysterious nature. Within comparatively recent times these ailments were set down as developments of "neurosis," and with that diagnosis all was at an end; little could be done for them. Now, however, the brilliant achievements of the surgery of the nervous system have made absolute cure possible in many cases, relief in nearly all. Trephining, exploration of the brain and the removal of tumours from it, and other operations have saved and restored to usefulness lives that seemed doomed to death or insanity. Patients are received at this hospital from all parts of the kingdom, and contributions from any district may be devoted to the maintenance of beds which will be specially allotted to patients coming from it, for the directors are exceedingly anxious that the institution should deserve to the full its title of "national" hospital.

THE following vacancies are announced this week, the amount of salary in each case being given after the name of the institution :—

**Resident Medical Officers.**

Bournemouth Friendly Societies Medical Association. Resident Medical Officer.—Salary £200, with residence and fees.
Menston Asylum, near Leeds. Medical Superintendent.—Salary £400 per annum, with board.
National Dental Hospital, Great Portland Street, W. House Surgeon.
Royal National Hospital for Consumption, Ventnor. Assistant Resident Medical Officer.
Royal Surrey County Asylum, Guildford. House Surgeon.—£80 per annum, with board.
Rubery Hill Asylum, Bromsgrove, Worcester. Clinical Assistant.—Board.
Western General Dispensary, Marylebone Road, N.W. Junior House Surgeon.—60 guineas per annum.
York Dispensary. Three Resident Medical Officers.—£130 per annum, with furnished apartments, etc.

**Non-resident Medical Officers.**

Bristol Royal Infirmary. Dental Surgeon.
Clapham General Dispensary. Medical Officer.
Downpatrick Union. Medical Officer, Killyleagh Dispensary.—£105 per annum, and fees.
Forest Hill Provident Dispensary. Medical Officer.
North-West London Hospital, Kentish Town Road. Assistant Physician.
St. John's Hospital for Diseases of the Skin, Leicester Square. Two Assistant Medical Officers.
St. Mary's Hospital for Women and Children, Quay Street, Manchester. Honorary Surgeon.

**Matron.**

Borough Fever Hospital, Sheffield.—£60.

**Nurses.**

Delancey Fever Hospital, Cheltenham. Night Nurse; not under 25.—Good salary if trained.
Memorial Hospital, Jarrow-on-Tyne. Two Trained Nurses—£20, with uniform.
Notts Nursing Institution. Hospital trained Medical, Surgical, and Monthly.—£25, increasing to £30, with indoor uniform.
Glasgow Maternity Hospital. Nurses
Workhouse Infirmary Nursing Association, 6, Adam Street, Adelphi, Strand Several trained.
Cancer Hospital, Myrtle Street, Liverpool Trained.
Nursing Institution, 96, Wimpole Street. Thoroughly trained.
Newport Infirmary, Monmouth. Charge Nurse.—£25, with uniform
Private Nurses' Home, 4, Stephenson Terrace, Preston, Lancashire. Medical, Surgical, and Monthly. Several.

**Probationers.**

Worthing Infirmary. One.

Newport Infirmary. One

---

NOT long ago *Punch* depicted a cabman who had been refused the excessive fare he asked, driving a hapless couple into the middle of a pond, and threatening to leave them there if his demand was not complied with. A somewhat similar spirit seems to actuate the workmen employed on the new small-pox hospital at Sheffield. Having contracted to work at it for 7½*d.* per hour they now demand 8½*d.*, and have gone on strike because it is refused them, trusting to the pressing necessity for the completion of the hospital to secure their request being granted. The honesty of such a course is more than doubtful, and its inhumanity entirely detestable.

---

"FAITH-HEALING," says the *Weekly Scotsman*, "is outdone by 'Christian science.' There is in Boston a lady named Eddy who conducts a medical school on a remarkable principle. For some trifling sum—say of £100—she undertakes to qualify a pupil in three weeks to perform what are called 'mind cures.' The patient is taught to abstract his or her mind from any disease or suffering. This effort of will makes you insensible even to a severe burn. There are people of credit who actually believe that bodily ills will depart in dudgeon if you only ignore them. Mr. Wemmick has only to say, 'Hallo, here's an attack of fever; let's take no notice of it,' and he will get well." But we should like to know how Mrs. Eddy's method succeeds in surgical cases, and also whether her mind-cures really cure diseases or only hypnotise the patient so as to make him unconscious of pain. There is a difference.

THE Bishop of Worcester has written a letter, which is to be circulated throughout his diocese, asking for contributions to the Worcester Infirmary. This institution has to face, at the same time, increased demands on its usefulness, and a decrease in its income amounting to £1,000 per annum. It has already begun to draw on its funded property, but as this is a suicidal fashion of living, it is to be hoped that the bishop's appeal will be promptly and liberally responded to.

---

OWING to the prevalence of small-pox at Sheffield the Great Northern and the Great Eastern Railway Companies have ceased running excursion trains into the town, and it is expected that this example will be followed by the other companies. Shopkeepers and merchants are suffering severely from the falling-off in the number of county customers, who are afraid to visit the town for the purpose of marketing. The Sheffield free libraries are still closed, and several private subscription libraries have also been temporarily suspended.

---

A DEPUTATION from the Council of the Royal Medica Benevolent College, consisting of Sir Edward Sieveking, Sir Joseph Fayrer, K.C.S.I., Dr. Holman, Dr. Galton, and several other gentlemen waited upon the Lord Mayor at the Mansion House, for the purpose of asking his Lordship to preside at the biennial festival dinner of the institution. Sir Edward Sieveking and Dr. Holman (the treasurer) having explained the objects of the College, and expressed the hope of the Council that they might secure influential assistance outside the medical profession, which could not be better done than by obtaining the aid of his Lordship, as representing the City of London, the Lord Mayor at once intimated his willingness to accede to the desire of the Council, and fixed Tuesday, the 17th of April, as the date of the festival.

---

COUNTRY districts might do worse than follow the example set by the "canny Scots" of Aberdeen. The parishes of Aberdeen, Old Aberdeen, Newhills, Nigg, and Banchory-Devenick have resolved to erect a combination hospital. The deputation which waited upon the convener of the Public Health Committee (Dr. Moir) in connexion with the scheme, expressed the readiness of each parish to pay £6 or £7 for each patient coming from their district treated in the hospital, or else to contribute a fixed annual sum. Dr. Moir said he considered the plan a feasible one. We venture to think that it is more than that; it is an eminently desirable one. If each parish in a district would undertake to give a certain sum annually to the support of a hospital in the neighbourhood, a great burden would be taken off the hospitals in our towns, and the sick and injured would no longer suffer from the long journeys to which they must now submit before reaching even the nearest hospital and infirmary.

---

THE January number of the *Medical Temperance Journal* contains a thoughtful paper by Mr. T. N. Kelynack, of Manchester, on "The Medical Man's relation to the Temperance Question," in which he points out and emphasises the fact that alcohol is a drug with a direct therapeutic action, and that its administration, like that of other drugs, should be left to the discretion of medical men; also that the reckless indulgence in alcoholic stimulants, which is the bane of our country, is an evil akin in character to the unregulated self-administration of opium and other poisons. One does not need to be an advocate of total abstinence to feel the truth of this; and conscientious medical men are very chary of recommending alcohol even as a drug, where any substitute equally good can be obtained, for fear of thus inducing a habit of inebriety. At the same time it must be made plain that alcohol is in very truth a valuable drug, and not, as teetotallers so often protest, merely a poison, and it is as plainly the practitioner's duty to prescribe it where he considers it necessary as to avoid recommending it to patients who would be equally benefited by another substance.

